# Human milk‐sharing practices and infant‐feeding behaviours: A comparison of donors and recipients

**DOI:** 10.1111/mcn.13389

**Published:** 2022-06-27

**Authors:** Jennifer A. Peregoy, Giovana M. Pinheiro, Sheela R. Geraghty, Katherine L. Dickin, Kathleen M. Rasmussen

**Affiliations:** ^1^ Division of Nutritional Sciences Cornell University Ithaca New York USA; ^2^ University of Cincinnati College of Medicine Cincinnati Ohio USA; ^3^ Department of Public and Ecosystem Health Cornell University Ithaca NY USA

**Keywords:** breastfeeding, human milk, infant feeding, lactation, milk sharing

## Abstract

Human milk sharing (HMS) is growing in popularity as an infant‐feeding strategy in the United States. HMS families are a hidden population because HMS is a nonnormative and stigmatized behaviour. Thus, gaining access to HMS participants is challenging, and research on this topic remains limited. In particular, little is known about the broader infant‐feeding behaviours of HMS parents. This study aimed to describe and compare the infant‐feeding behaviours and HMS practices among a network of HMS donors and recipients. A detailed online survey was distributed to HMS parents in the Washington, DC region. Bivariate analyses were used to summarize the data by donor/recipient status when possible. Group differences were tested using analysis of variance for continuous variables and *χ* ^2^ tests for categorical variables. Donors and recipients did not differ in their sociodemographic characteristics. Recipients were significantly more likely than donors to have experienced complications of labour and delivery, traumatic birth, postpartum depression or a negative breastfeeding experience. Donors and recipients did not differ significantly in their duration of lactation or HM‐feeding. Interestingly, 30% of recipients ever produced excess milk and 21% of donors ever had difficulty producing enough milk for their child. Compared with donors, recipients faced numerous maternal health challenges, but were still able to achieve a long duration of HM‐feeding. HMS recipients represent a vulnerable group who may benefit from additional psychosocial and lactation support to improve their health and breastfeeding outcomes. Additional research is needed to investigate the associations between HMS participation, infant‐feeding behaviours and lactation outcomes.

## Key messages

- All human milk sharing (HMS) participants achieved a long duration of HM‐feeding, reflecting a high value placed on HM and a strong commitment to HM‐feeding.
- Recipients in this sample were largely using HMS as a strategy to supplement the mother's own milk.
- Approximately one‐third of recipients ever produced more HM than needed and one‐fifth of donors ever had difficulty producing enough milk, suggesting that *both* donors and recipients experienced breastfeeding challenges.
- Many HMS recipients encountered compounded maternal medical and mental health challenges and would therefore benefit from additional psychosocial and lactation support to improve their mental health and breastfeeding outcomes.

## 1 INTRODUCTION

Human milk (HM) provides numerous health benefits for both infant and mother (Dewey et al., 1995; Ip et al., 2007; Kramer et al., 2007). Public health and medical authorities recommend that infants be exclusively breastfed for 6 months, with continued breastfeeding for 1 year or longer (Eidelman, 2012; Gartner et al., 2005; Kramer & Kakuma, 2012; Martens, 2012). However, many American women fall short of meeting those goals (Prevention, 2020) and, thus, rely on various supplemental feeding strategies to feed their infants. HM sharing (HMS) has recently emerged as one such supplemental infant‐feeding strategy. This study defines HMS as the commerce‐free exchange of HM between individuals for infant feeding that occurs outside of the formal milk banking system. HMS is sometimes referred to as peer to peer or informal because it relies on the individual participants to negotiate the specific terms of the exchange (A. E. L. Palmquist et al., 2019). It is important to note that this phenomenon is distinct from HM selling, which is also done peer to peer, but includes a financial transaction. HMS is a contemporary transformation of historical allonursing—one that still relies on a donor with surplus milk and a recipient with a milk deficiency, but is no longer limited by temporality and geographic proximity because of the modern inventions of refrigeration, the internet and the double‐electric breast pump.

Contemporary HMS has been largely enabled by the ready availability of surplus expressed HM (E‐HM). In the industrialized, high‐income populations where HMS has been studied, mothers rely substantially on HM expression to feed their own infants (Boswell‐Penc & Boyer, 2007; Labiner‐Wolfe et al., 2008). This has created an environment ripe for HM exchange, where some women are unable to produce enough milk to feed their infants and others produce in excess, sometimes accumulating sizable quantities of E‐HM in their freezers. The key players of HMS are donors and recipients. Donors are the individuals who produce and donate their HM in an HMS arrangement, while donor families are the entire family unit of the donor parent, including the donor children (the children who would have consumed the HM, had it not been shared with another family). Recipients are the adult individuals who receive the shared HM (S‐HM) with the intention of feeding it to children under their care (who may or may not be their parents). Recipient families include the entire family unit of the recipient, and recipient children are the children who ultimately consume the S‐HM.

In the United States, HMS donors are sociodemographically similar to the women who donate to Human Milk Banking Association of North America & Association (HMBANA) milk banks—married, healthy, White, well‐educated and financially secure (Osbaldiston & Mingle, 2007). HMS recipients are often mothers with a strong desire to breastfeed, but who experience breastfeeding challenges and lactation insufficiency (Cassar‐Uhl & Liberatos, 2018; McCloskey & Karandikar, 2019; A. E. Palmquist & Doehler, 2014; Perrin et al., 2014; Tomori et al., 2016). Parents whose infants are experiencing inadequate growth or are intolerant of infant formula are another important subgroup of HMS recipients (Cassar‐Uhl & Liberatos, 2018; McCloskey & Karandikar, 2018; O'Sullivan et al., 2018; A. E. Palmquist & Doehler, 2014).

The self‐regulated and peer‐to‐peer nature of HMS renders it a highly individualized and heterogeneous set of practices. Prior research has shown that much of HMS is facilitated through the internet, where donors and recipients connect using Facebook groups and milk‐sharing websites (Akre et al., 2011; Keim et al., 2014; O'Sullivan et al., 2018; A. E. Palmquist & Doehler, 2014; Perrin et al., 2014; Palmquist & Doehler, 2015). Less is known about milk sharing that operates at a local level among friends, family and community members. Some HMS recipients rely on one or two long‐term donors, while others receive S‐HM from many different donors (Gribble, 2014; A. E. Palmquist & Doehler, 2014; Reyes‐Foster et al., 2015; Thorley, 2008). An important area of interest to specialists in maternal and child nutrition is learning how S‐HM is incorporated into the overall infant‐feeding strategies of HMS families. This remains an under‐researched topic within the HMS literature.

HMS has generated significant controversy because of the potential risks involved and the “yuk factor,” as the notion of feeding one woman's milk to another's child may generate feelings of disgust or aversion (Shaw, 2004, 2007). Microbial and viral pathogens can be transmitted in HM (Ando et al., 2004; Bardanzellu et al., 2018; Bowen et al., 2017; Josephson et al., 2014; Sosa & Barness, 1987). HM can also be contaminated with a prescription or recreational drugs, or altered by suboptimal practices during expression or storage that could lead to microbial contamination or loss of nutrients (Burra et al., 2019; Datta et al., 2019; Eglash et al., 2017; Fierro et al., 2019; Hamosh et al., 1996, 1997). Given these potential risks, numerous organizations, including the US Food and Drug Administration and HMBANA, have released statements cautioning against HMS and positioning it as a high‐risk behaviour (HMBANA & European Milk Bank Association, 2015; United States Food and Drug Administration, 2015). Yet, the common alternative to maternal milk, infant formula, also has numerous risks associated with it, and mothers are keenly aware of these risks (Gribble, 2012). It is within this fraught context that the practice of milk sharing has continued, underscoring the strong demand for HM (Akre et al., 2011; Keim et al., 2014; O'Sullivan et al., 2018) and highlighting the importance of expanding research on this increasingly prevalent infant‐feeding strategy. Gaps in the HMS scientific literature are numerous because this study is still in its infancy.

The primary objective of this study was to describe the infant‐feeding behaviours and HMS practices among a geographically defined network of milk‐sharing parents in the greater Washington, DC metropolitan region. In addition, we aimed to identify differences between donors and recipients in their maternal health characteristics, infant‐feeding behaviours and HMS practices.

## 2 METHODOLOGY

### 2.1 Setting

This study used a cross‐sectional web‐based survey. The study was conducted during July 2019–May 2020 in the greater Washington, DC metropolitan region (DMV, which comprises the District of Columbia and parts of Maryland and northern Virginia). According to the 2020 US Census, the DMV region has an estimated total population of 6.4 million and is a highly affluent and well‐educated population (Hess, 2018; Martin, 2019). According to the most recently available data, 88% of infants born in the District of Columbia in 2017 were ever breastfed, 65% were still breastfeeding at 6 months and 24% were exclusively breastfed for 6 months (Centers for Disease Control and Prevention, 2020).

*Human Milk 4 Human Babies* and *Eats on Feets* are the two primary organizations that facilitate the bulk of online HMS by hosting region‐specific Facebook groups, providing fora for donors and recipients to connect. The DMV region has two active *Human Milk 4 Human Babies* Facebook groups, with just over 22,000 cumulative followers, and three active *Eats on Feets* Facebook groups, with approximately 7200 cumulative followers. These groups are highly active, suggesting a robust network of HMS parents in the DMV region.

### 2.2 Sample

Convenience sampling was used to reach members of the target population of milk‐sharing parents in the DMV region. Study inclusion criteria were as follows: aged 18 years or older, English‐speaking, had engaged in milk sharing in the past 18 months (as a donor or recipient) and lived or worked in the DMV region at the time of milk sharing. Parents were recruited through a variety of convenience sampling techniques, namely, posting advertisements on local milk‐sharing websites and parenting listservs, sharing recruitment materials with local birth workers (lactation consultants, doulas and midwives) to share with their clients and snowball sampling.

### 2.3 Measurement

The survey questionnaire was informed by the findings of a previously conducted ethnographic study with HMS recipients (Peregoy, 2021). Those semistructured interviews with HMS recipients provided detailed information about how HMS is organized and practiced. This information was used to develop broad content themes and guide the design of the questions and response choices. The survey tool was reviewed for content by research colleagues and validated by members of the target population. The validation study (*n* = 11) used cognitive interviewing to assess construct validity and to refine the survey questions for improved clarity and validity. Finally, usability testing (*n* = 8) was conducted to identify and correct issues with the web‐based version of the survey before commencing data collection. The study team made real‐time modifications to the survey during the validation study and usability testing in an iterative process of fine‐tuning and improvement.

### 2.4 Data collection

The online survey link was distributed through the various channels described above. Parents interested in participating first completed a brief screening questionnaire to ascertain eligibility. Eligible respondents were then provided with the informed consent script, which they had to read and consent to proceed with the survey. Among respondents, the mean survey completion time was 37 min. Within 48 h of survey completion, all eligible respondents received a participation incentive in the form of a $20 Amazon gift card. The convenience sampling yielded 168 respondents, 58% donors and 42% recipients.

### 2.5 Data analysis

All data cleaning, recoding and analyses were performed using SAS Studio version 9.04. Bivariate analysis of sociodemographic factors, infant‐feeding behaviours and HMS practices was conducted by donor/recipient (D/R) status for all survey items asked of both donors and recipients. Differences by D/R status in continuous variables were tested using analysis of variance and in categorical measures using *χ* ^2^ tests or Fisher's exact tests for small cell sizes. For nonnormally distributed variables, data were reported as medians with interquartile range and significance testing was conducted using Wilcoxon's rank test.

## 3 RESULTS

### 3.1 Sample characteristics

Nearly all respondents self‐identified as women (Table 1). Sex data were not available. Therefore, female gender pronouns will be used in describing this sample. Donors and recipients did not differ in their sociodemographic profiles. However, the age distribution of recipients skewed older than donors (*p* < 0.05). The majority of the women in the sample were non‐Hispanic White, married, employed and highly educated.

**Table 1:** Sociodemographic characteristics of the study participants (*n* = 168), stratified by HMS donor/recipient status

	Recipients (*n* = 70)		Donors (*n* = 98)		Total (*n* = 168)	
Sociodemographic characteristic	*N*	%	*N*	%	*N*	%
Current age*						
18–34 years	33	48.6	62	67.4	95	59.4
35–44 years	34	50.0	30	32.6	64	40.0
45–54 years	1	1.5	0	0.0	1	0.6
Self‐identified gender						
Woman	70	100.0	91	98.9	161	99.4
Racial/ethnic background						
White	61	88.4	77	83.7	138	85.2
Black	0	0.0	3	3.3	3	1.9
Asian	4	5.8	7	7.6	11	6.8
Latino/Hispanic	2	2.9	3	3.3	5	3.1
Multiethnic or other	2	2.9	2	2.2	4	2.5
Marital status						
Single/never married	2	2.9	2	2.2	4	2.5
Married/domestic partnership	68	97.1	90	97.8	158	97.5
Partner's gender identity						
Man	62	88.6	87	94.6	149	92.0
Woman	5	7.1	2	2.2	7	4.3
Nonbinary	1	1.4	1	1.1	2	1.2
Highest level of education completed						
Associate's degree/some college	5	7.1	4	4.3	9	5.6
Bachelor's degree	22	31.4	27	29.3	49	30.2
Master's degree	34	48.6	48	52.2	82	50.6
Doctoral‐level degree	9	12.9	13	14.1	22	13.6
Current employment status						
Unemployed—full‐time parent	11	15.7	15	16.3	29	17.9
On parental leave	4	5.7	2	2.2	6	3.7
Employed part‐time	12	17.1	9	9.8	21	13.0
Employed full‐time	43	61.4	65	70.7	108	66.7
Estimated annual household income						
<$49,999	4	5.7	2	2.2	6	3.7
$50,000–$99,999	12	17.1	11	12.0	23	14.2
$100,000–$149,999	17	24.3	21	22.8	38	23.5
$150,000–$199,999	11	15.7	21	22.8	32	19.8
$200,000–$299,999	15	21.4	28	30.4	43	26.5
$300,000 or more	11	15.7	9	9.8	20	12.3

Abbreviation: HMS, human milk sharing.

* *p* < 0.05.

Women in the sample had a mean of 1.6 liveborn children (median: 1, interquartile range [IQR]: 1–2; no difference by D/R status). Several pregnancy and birth characteristics differed by D/R status (Table 2). Specifically, a three‐fold higher percentage of recipients experienced labour and delivery complications compared with donors (*p* < 0.0001). Recipients were approximately twice as likely as donors to report that the birth was a traumatic experience for them (*p* < 0.05) and approximately three times as likely as donors to have experienced postpartum depression (*p* < 0.0001).

**Table 2:** Maternal and child health characteristics of the study participants (*n* = 168), stratified by HMS donor/recipient status

	Recipients (*n* = 70)		Donors (*n* = 98)		Total (*n* = 168)	
Maternal and child health characteristic	*N*	%	*N*	%	*N*	%
Singleton child*	66	94.3	98	100	164	97.6
Primiparous	37	53.6	51	52.0	88	52.7
Maternal age at the youngest child's birth*						
Less than 18 years old	4	5.7	3	3.1	7	4.2
18–29 years	7	10.0	12	12.2	19	11.3
30–34 years	29	41.4	62	63.3	91	54.2
35–39 years	25	35.7	21	21.4	46	27.4
40 years or older	5	7.1	0	0.0	5	3.0
Maternal health complications						
Had complications during pregnancy	17	24.3	15	15.3	32	19.0
Had complications during labour/delivery**	26	37.1	11	11.2	37	22.0
Considered the birth traumatic*	24	35.3	18	18.6	42	25.5
Ever experienced PP depression**	26	38.2	13	13.4	39	23.6
Ever experienced PP anxiety	28	41.2	39	40.2	67	40.6
Gestational age at birth						
28–31 weeks	2	2.9	3	3.1	5	3.0
32–36 weeks	5	7.1	7	7.1	12	7.1
37+ weeks	63	90.0	88	89.8	151	89.9
Caesarean delivery	23	32.9	21	21.4	44	26.2
Employed at the time of child's birth	60	85.7	84	85.7	144	85.7
Parental leave situation						
I reduced my hours or took unpaid leave	6	10.0	15	17.9	21	14.6
I took partial or fully paid leave	49	81.7	61	72.6	110	76.4
I stopped working	4	6.7	5	6.0	9	6.3
Parental leave duration						
1–7 weeks	5	8.6	5	6.2	10	7.2
8–11 weeks	8	13.8	13	16.0	21	15.1
12–15 weeks	27	46.6	34	42.0	61	43.9
16–23 weeks	15	25.9	20	24.7	35	25.2
24+ weeks	3	5.2	9	11.1	12	8.6
Recipient child health characteristics						
Child had a health issue (currently or in the past)	11	16.0	‐	‐	‐	‐
Child had a dietary allergy, sensitivity or intolerance (currently or in the past)	12	17.4	‐	‐	‐	‐
Child was diagnosed with tongue and/or lip tie	23	33.3	‐	‐	‐	‐

Abbreviations: HMS, Human milk sharing; PP, post‐partum.

* *p* < 0.05

** *p* < 0.0001.

Recipients were asked a series of questions about the children for whom they were obtaining milk. The mean age of the recipients' children at the time of the survey was 14 months (SD: 12.2; median: 11.3, IQR: 6–20), and the mean age at which they began consuming S‐HM was 4.6 months (SD: 4.0; median: 4.0, IQR: 0.8–8.0). Notably, one‐third of the recipients' infants had been diagnosed with a tongue and/or lip tie and 78% of those children had the tongue/lip tie surgically released at a mean age of 4.8 weeks.

### 3.2 Breastfeeding experience and infant‐feeding behaviours

**Figure 1:**
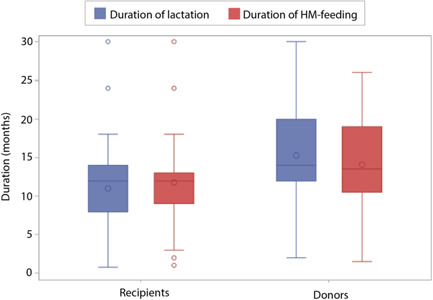
Comparison of the duration of the most recent lactation and duration of human milk (HM) feeding (months) of the youngest child among HM‐sharing donors and recipients who are no longer feeding HM to their children (*n* = 58). The boxes represent the interquartile range containing the central 50% of values; the lines across the box represent the median values; the circles in the box represent the mean values; the whiskers represent the “minimum” (Q1 − 1.5 × IQR) and “maximum” (Q3 + 1.5 × IQR); and the circles represent outliers.

Respondents were asked a series of questions about their breastfeeding experience. Among respondents who were no longer feeding their children HM (*n* = 58), the median duration of HM feeding did not differ significantly between donors (13.5 months, IQR: 10.5–19.0) and recipients (12.0 months, IQR: 9.0–13.0; *p* = 0.1334) (Figure 1). In their lifetime of lactation experience, recipients were less likely than donors to have ever produced more milk than needed by their child (*p* < 0.0001) and more likely to have ever had difficulty producing enough milk for their child (*p* < 0.0001), been diagnosed with a health problem affecting lactation (*p* < 0.05) or to have fed infant formula to their child (*p* < 0.05) (Table 3). Overall, 29% of respondents reported that they had ever exclusively pumped to feed their child (which did not differ significantly by D/R status). Interestingly, 30% of recipients ever produced more HM than they needed and 21% of donors ever had difficulty producing enough milk for their child. Nearly half of the donors rated their most recent breastfeeding experience as very positive, while recipients were significantly more likely to rate their most recent breastfeeding experience negatively (29%; *p* < 0.05).

**Table 3:** Breastfeeding experience and infant‐feeding behaviours of study participants (*n* = 168), stratified by HMS donor/recipient status

	Recipients (*n* = 70)		Donors (*n* = 98)		Total (*n* = 168)	
Breastfeeding or infant‐feeding behaviour	*N*	%	*N*	%	*N*	%
For any of their children, had ever:						
Produced HM	66	94.3	97	99.0	163	97.0
Nursed directly at the breast	64	97.0	96	99.0	160	98.2
Pumped milk to feed their child	66	100.0	94	96.9	160	98.2
Exclusively pumped to feed their child	24	36.4	22	23.4	46	28.8
Had difficulty producing enough HM**	54	81.8	20	20.6	74	45.4
Produced more HM than needed**	20	30.3	90	92.8	110	67.5
Been diagnosed with a health problem that affected lactation*	13	18.6	3	3.1	16	9.5
Fed infant formula to their child*	50	71.4	43	43.9	93	55.4
IFP used for the child of most recent lactation						
Child has ever received infant formula**	45	64.3	32	32.7	77	45.8
Child is currently receiving infant formula	4	5.7	4	4.1	8	4.8
Child is currently receiving HM	40	57.1	68	69.4	108	64.3
IFP used during the first 3 months for the child of most recent lactation						
Feeding at the breast of a nursing parent	62	88.6	93	94.9	155	92.3
Nursing parent's own E‐HM	52	74.3	72	73.5	124	73.8
S‐HM**	40	57.1	1	1.0	41	24.4
Commercial infant formula*	29	41.4	23	23.5	52	31.0
B‐HM*	11	15.7	1	1.0	12	7.1
Overall, how do you feel about your breastfeeding experience with your youngest child?*						
Very negative	4	6.2	0	0	4	2.48
Somewhat negative	15	23.1	7	7.3	22	13.7
Neutral	11	16.9	5	5.2	16	9.9
Somewhat positive	24	36.9	36	37.5	60	37.3
Very positive	9	13.9	46	47.9	55	34.2

Abbreviations: B‐HM, banked human milk; E‐HM, expressed human milk; HM, human milk; IFP, infant‐feeding practice; S‐HM, shared human milk.

* *p* < 0.05

** *p* < 0.0001.

### 3.3 Milk‐sharing practices

Very few respondents had ever sold (0.6%) or purchased (2.4%) HM (Table 4). Recipients were significantly more likely than donors to have ever received banked HM (B‐HM; *p* < 0.05). Twenty‐four percent of recipients had ever donated S‐HM and 20% had donated S‐HM in the past 18 months, while just 3.1% of donors had ever received S‐HM.

**Table 4:** Milk‐sharing practices of study participants (*n* = 168), stratified by HMS donor/recipient status

	Recipients (*n* = 70)		Donors (*n* = 98)		Total (*n* = 168)	
Milk‐sharing practice	*N*	%	*N*	%	N	%
Prevalence of *receiving* human milk						
Has ever purchased HM^a^	1	1.4	3	3.1	4	2.4
Has ever received HM from a milk bank*	13	18.6	5	5.1	18	10.7
Has ever had their baby cross‐nursed by another person*	3	4.3	4	4.1	7	4.2
Has ever received S‐HM**	70	100	3	3.1	73	43.5
Has received S‐HM in the past 18 months**	70	100	0	0	70	41.7
Prevalence of *providing* human milk						
Has ever sold HM^a^	0	0	1	1.0	1	0.6
Has ever provided HM to a milk bank^a^	1	1.4	3	3.1	4	2.4
Has ever cross‐nursed another person's baby*	4	5.7	3	3.1	7	4.2
Has ever donated S‐HM**	17	24.3	98	100	115	68.5
Has donated S‐HM in the past 18 months**	14	20.0	81	82.7	95	56.5
Methods used for connecting with HMS parents						
Online group (e.g., EOF, HM4HB, BF listserv, etc.)	39	55.7	59	60.2	98	58.3
I already knew them**	5	7.1	42	42.9	47	28.0
Facilitated through a mutual friend/acquaintance*	3	4.3	15	15.3	18	10.7
Facilitated through a lactation consultant*	12	17.1	5	5.1	17	10.1
Facilitated through a midwife or doula**	36	51.4	4	4.1	40	23.8
Ever shared milk with the following individuals:						
Friend	47	67.1	33	33.7	80	47.6
Online acquaintance that you *have not* met in person	21	30.0	36.	36.7	57	33.9
Online acquaintance that you *have* met in person*	27	38.6	22	22.4	49	29.2
Someone you connected with through an intermediary	7	10.0	13	13.3	20	11.9
Family member	9	12.9	8	8.2	17	10.1
Someone you met in your local community (offline)	2	2.9	5	5.1	7	4.2
Only shared milk with friends and/or family*	30	42.9	25	25.5	55	32.7
Methods of milk exchange						
Directly (met in person to pick up milk)*	66	94.3	82	83.7	148	88.1
Indirectly (milk was given to someone else to give to the recipient)	17	24.3	21	21.4	38	22.6
Received via mail/shipped^a^	3	4.3	1	1.0	4	2.4
Via cross‐nursing^a^	0	0	2	2.0	2	1.2
Type of milk donated^a^						
Surplus E‐HM originally intended to feed my child	*‐*	*‐*	70	72.2	‐	‐
HM that I expressed specifically for donating	*‐*	*‐*	5	5.2	‐	‐
Both surplus E‐HM and HM expressed for donating	*‐*	*‐*	23	23.7	‐	‐
Estimated proportion of child's HM intake that was S‐HM (during HMS arrangement)^a^						
A little or some	25	36.2	‐	‐	‐	‐
About half	17	24.6	‐	‐	‐	‐
Most or all	22	31.9	‐	‐	‐	‐
It varied	5	7.2	‐	‐	‐	‐
Child was receiving mother's own milk while milk sharing^a^	59	85.5	‐	‐	‐	‐
Child was still feeding at the mother's breast during part or all of milk‐sharing arrangement^a^	49	71.0	‐	‐	‐	‐

Abbreviations: BF, breastfeeding; E‐HM, expressed human milk; EOF, Eats on Feets; HM, human milk; HM4HB, Human Milk 4 Human Babies; HMS, human milk sharing; S‐HM, shared human milk.

^a^ Statistical testing was not conducted due to small cell sizes or lack of a comparison group.

* *p* < 0.05

** *p* < 0.0001.

Donors reported donating their milk to a mean of 2.3 recipients (median: 2.0, IQR: 1–3); on average, multiple donations were made to 0.9 people (median: 1.0, IQR: 0–1). This compares to recipients, who reported receiving S‐HM from a mean of 3.4 donors (median: 1.0, IQR: 1–4); 1.3 of these were persons who donated on more than one occasion (median: 1.0, IQR: 0–1). HMS recipients in this sample milk shared for an average duration of 3.3 months (median: 2.0, IQR: 0.5–4.8).

The majority of respondents reported initially connecting with HMS parents through an online group (Table 4). Recipients were more likely than donors to have had a midwife or doula facilitate their connection to HMS parents (*p* < 0.0001) and to have *only* milk shared with friends or family (42.9% vs. 25.5%, respectively; *p* < 0.05). S‐HM was primarily exchanged directly in person, with less than a quarter of respondents having exchanged milk indirectly via a facilitator, and very few having exchanged milk via mail. Nearly three‐quarters of donors only donated HM that was expressed with the intention of feeding their own children. While milk sharing, 86% of recipient infants were still consuming their mother's own milk and 71% were still feeding at their mother's breast during part or all of the HMS arrangement.

The estimated total volume of S‐HM exchanged did not differ by D/R status (Supporting Information: Figure 1). Fifty‐five percent of respondents exchanged a total of less than 250 ounces of S‐HM, while 22% exchanged 1000 or more ounces.

## 4 DISCUSSION

This study makes an important contribution to the literature by describing in detail the HMS practices and infant‐feeding behaviours among a network of HMS parents in a large American metropolitan region. We found that all HMS participants achieved a long duration of HM‐feeding and used a variety of infant‐feeding behaviours, including at‐the‐breast feeding, formula feeding, milk sharing and exclusive pumping. We also found that, although donors and recipients did not differ in sociodemographic characteristics, they differed in their maternal experiences and some infant‐feeding behaviours.

Although efforts were made to recruit a diverse sample by ethnicity, socioeconomic status and milk sharing type (online vs. community‐based); ultimately, this was a homogeneous sample of non‐Hispanic White, highly educated, married and employed women of high socioeconomic status. Thus, our sample composition mirrors that of other HMS studies (Cassar‐Uhl & Liberatos, 2018; Palmquist & Doehler, 2014; Paynter & Goldberg, 2018; Perrin et al., 2016; Reyes‐Foster et al., 2015) and reflects the characteristics of mothers with the highest rates of breastfeeding in the United States (Fein et al., 2008). It remains unknown if this is a representative sample of the population of HMS parents or if we and others have done an inadequate job of finding and including the full range of HMS participants. Previous results from a large online survey of online HMS participants showed that donors reported higher income and educational attainment than recipients (A. E. Palmquist & Doehler, 2014). However, in our smaller geographically defined sample, donors and recipients did not differ in these characteristics.

The significant differences between recipients and donors in their birth and postpartum experiences are relevant to understanding the potential behavioural drivers and needs of these groups. HMS recipients were more likely than donors to have experienced complications of labour and delivery, a traumatic birth, postpartum depression or an overall negative experience with breastfeeding. In previous research, a higher proportion of recipients had caesarean deliveries and preterm births than donors (A. E. Palmquist & Doehler, 2014), but this was not the case in our study. Interestingly, a high proportion of both donors and recipients (41%) in our sample reported experiencing postpartum anxiety, underscoring the pressures on busy working mothers who are juggling competing responsibilities. Together, these findings indicate that HMS recipients are a vulnerable group of women, many of whom encountered compounded medical and mental health challenges and require additional psychosocial and lactation support to improve both their mental health and their breastfeeding outcomes.

This study revealed interesting lactation experiences among HMS participants. Both recipients and donors reported a long duration of most recent lactation, reflecting a strong commitment to breastfeeding in this population, corroborating previous research (A. E. Palmquist & Doehler, 2014). It is noteworthy that despite experiencing numerous maternal health challenges and breastfeeding issues, recipients were able to achieve a duration of HM feeding that did not differ significantly from donors. It is likely that receiving S‐HM was an important strategy that helped them to achieve their HM‐feeding goals. Another interesting finding is that 21% of donors ever had difficulty producing enough milk and 30% of recipients ever produced more milk than needed. Furthermore, 20% of HMS recipients had also donated their milk in the last 18 months. These findings suggest that *both* recipients and donors experienced challenges during their breastfeeding journeys, underscoring the complexity and mutable nature of the breastfeeding journey. Women may serve as both donors and recipients during a given lactation period, implying the potential for crossover between donor and recipient status, as supported by previous research findings (Reyes‐Foster et al., 2015). These findings suggest that donor/recipient status is transient, with more nuance and complexity to the HMS experience than previously assumed by the research community.

Overall, 45% of all respondents indicated that they ever had difficulty producing enough milk, which raises questions about actual versus perceived lactation insufficiency. Perceived lactation insufficiency is common among breastfeeding women and is associated with breastfeeding discontinuation and nonexclusivity (Gianni et al., 2019; Hillervik‐Lindquist, 1991; Hillervik‐Lindquist et al., 1991; Mathur & Dhingra, 2009; Neifert & Bunik, 2013; Sandhi et al., 2020). Additional research is warranted to investigate the role that perceived lactation insufficiency plays in milk sharing.

A higher proportion of our respondents had exclusively pumped (28.8%) than was reported in another US population (6.9%) (Keim et al., 2017). It seems logical that exclusive pumping is a common behaviour among this population, given that contemporary HMS requires a steady supply of surplus E‐HM, which is enabled by the use of breast pumps. It is relatively easy to appreciate why a mother who is experiencing breastfeeding challenges—latch issues, in particular—might turn to exclusive pumping to feed her infant and then to receiving S‐HM. However, the proportion of donor mothers who have exclusively pumped was not significantly lower and the motivations are less obvious for this group. It is likely that some HMS donors use exclusive pumping as a strategy to manage excessive supply, a phenomenon that has been documented in previous research (Gribble, 2014). Further research is warranted to understand the role that exclusive pumping plays in HMS, as well as the reasons why parents turn to exclusive pumping, another breastfeeding topic with a paucity of data.

The finding that one‐third of recipients' infants had been diagnosed with tongue/lip tie is notable, as this is substantially higher than the reported prevalence range among the general population of infants (4%–10%) (Hill et al., 2021; Segal et al., 2007). Thus, tongue/lip tie could be viewed as a potential causal factor for the breastfeeding challenges experienced by this group. However, between 1997 and 2012, the diagnosis of tongue/lip tie has increased more than 800% nationally (Walsh et al., 2017), and its diagnosis remains a controversial topic without clear agreement on best practices (Fraser et al., 2020; LeFort et al., 2021; Unger et al., 2020). Additionally, a Cochrane review of the effect of frenotomy on infant feeding found that performing frenotomy reduced breastfeeding mothers' nipple pain, but did not have a consistent positive effect on infant breastfeeding outcomes (O'Shea et al., 2017). Thus, our findings of a high prevalence of tongue/lip tie among HMS recipients must be interpreted with caution, as neither its diagnosis nor frenotomy procedures have clear implications for infant‐feeding behaviours and breastfeeding outcomes. An additional possibility is that the infants in this sample may have been misdiagnosed with tongue/lip tie, resulting in an unresolved underlying breastfeeding issue. This remains an area of infant feeding in need of high‐quality research to deepen our understanding of the condition, its management and its impact on infant‐feeding outcomes.

Recipients in this sample were largely using HMS as a strategy to supplement the mother's own milk, with the majority of recipients still feeding their infants at the breast during the HMS arrangement. Furthermore, the mean age at which the recipient children began consuming S‐HM was 4.6 months, with a mean HMS participation duration of 3.3 months. Taken together, these data paint a picture of HMS among this sample as a temporary supplemental infant‐feeding strategy for healthy, partially to predominantly breast milk‐fed infants. However, this also means that 14% of infants were no longer receiving mother's milk when milk sharing occurred, and 29% were no longer feeding at their mother's breast. These findings suggest that there is a nontrivial faction of HMS recipients who were unable to overcome their breastfeeding difficulties and were likely in need of additional support. Unfortunately, to our knowledge, there are no other published studies with which to compare these findings.

It has been suggested that the emergence of HMS has negatively affected milk banks by competing for the same pool of eligible donors, thus reducing the supply of B‐HM available for milk banks and neonatal intensive care units (Dutton, 2011; Jones, 2013; Newman, 2011; Rochman, 2011). However, our results highlighted characteristics of recipient children and donors that suggest that HMS participants would not have been eligible to receive HM from or donate it to HMBANA milk banks. The recipient children in this study were primarily healthy, full‐term babies who began to receive S‐HM at a mean age of 4.6 months. The modest HMBANA milk supply is typically reserved for preterm, sick and vulnerable infants. Therefore, it is unlikely that the recipients in this study would have been eligible to receive B‐HM. The majority of the HMS donors in this study also would not have been eligible for milk bank donation, given that HMBANA milk banks require donors to complete a detailed screening process *before* expressing the milk to be donated. Our findings are supported by previous research showing that donors and recipients would not have been eligible for milk bank donation (Gribble, 2013). Taken together, these findings suggest that the practice of HMS does not compete with HM banks because these two types of donated HM serve different needs among distinct groups.

Study participants engaged in certain HMS practices with important implications for the quality of S‐HM. First, we found that the S‐HM was predominantly exchanged directly between donors and recipients, eliminating the risks posed by shipping HM (e.g., temperature dysregulation, microbial growth and leakage). Second, the majority of donors in this study donated E‐HM originally intended to feed their own children, suggesting that the S‐HM *quality* is likely to be similar to that of the E‐HM that mothers are feeding to their own children. Indeed, a recent study analysed samples of S‐HM, B‐HM and E‐HM expressed for a mother's own infant and found no difference in the rates of total aerobic bacterial or coliform growth, lysozyme activity, sIgA activity, lactose, fat, protein or water content between the samples (Perrin et al., 2018). Taken together, these findings suggest that, although mothers may not consistently follow HM handling and storage guidelines (Carre et al., 2018; Reyes‐Foster et al., 2017), the quality of S‐HM may be similar to that of E‐HM that mothers are feeding their own infants.

### 4.1 Strengths and limitations

This study has several notable strengths. The development of the survey tool was informed by the findings of a detailed ethnographic study with HMS recipients, resulting in a survey tool closely aligned with the lived experiences of milk‐sharing parents. The survey tool was then subjected to two rounds of cognitive testing and refinement to ensure its construct validity. Another strength is the highly detailed survey tool, which covered a range of experiences and behaviours. Thus, the survey tool is the core strength of this study. Additionally, sampling was conducted using various outreach methods to ensure that both online and community‐based HMS participants were adequately represented in this study.

There are several limitations to this study. The use of nonrandom sampling techniques introduced potential bias into the sample. Our reliance on online recruitment strategies likely underrepresented HMS parents who do not use the internet to connect with other parents. Therefore, the data from this survey cannot be extrapolated to the general population of milk‐sharing parents. Furthermore, because the survey was limited to HMS participants in the DMV region, these findings are limited in their generalizability to other geographic settings. The study inclusion criterion of having milk shared within the past 18 months introduces the potential for recall bias among parents who milk shared many months previously, likely during a stressful and sleep‐deprived period in their lives. However, this was minimized in our study because 74% of respondents had milk shared in the 9 months before taking the survey. Finally, the sample size of this study limited our ability to test for statistical differences in less commonly reported HMS practices.

## 5 CONCLUSION

This study adds to the body of knowledge of milk sharing by describing the infant‐feeding behaviours and milk‐sharing practices among a US network of milk‐sharing parents in detail and identifying important differences and similarities between HMS donors and recipients. We found that overall, HMS participants are achieving a long duration of lactation and HM feeding and, although not sociodemographically different, donors and recipients differed significantly in their maternal experiences and some infant‐feeding behaviours. Many HMS recipients encountered compounded medical and mental health challenges, and thus represent a vulnerable population in need of additional psychosocial and lactation support to improve their mental health and breastfeeding outcomes. Additional research is needed to further elucidate how HM sharing is incorporated into and modifies broader infant‐feeding patterns.

## AUTHOR CONTRIBUTIONS

Jennifer A. Peregoy conceptualized and designed the online survey instrument, conducted the statistical analysis and drafted the first version of the manuscript. Giovana M. Pinheiro assisted with the conceptualization and design of the survey instrument, as well as its validation. Sheela R. Geraghty assisted with the drafting and editing of the manuscript. Katherine L. Dickin assisted with the design of the survey tool and editing of the manuscript. Kathleen M. Rasmussen made substantial contributions to the design of the survey instrument and the statistical analysis plan and assisted with all stages of manuscript preparation. All authors approved the final manuscript as submitted.

## CONFLICT OF INTEREST

The authors declare no conflict of interest.

## ETHICS STATEMENT

This study was evaluated by Cornell University's Institutional Review Board before its initiation and was granted exempt status.

## Supporting information

Supplementary information.

## Data Availability

Data are available on request due to privacy/ethical restrictions.
