# Supplementary material for: Human milk‐sharing practices and infant‐feeding behaviours: A comparison of donors and recipients
Source: Matern Child Nutr. 2022 Jun 27;18(4):e13389. doi: 10.1111/mcn.13389 (PMC9480963; doi:10.1111/mcn.13389)
Supplement: Supplementary file 1 — Supplementary information. [file MCN-18-e13389-s001.docx]

**Supplemental Files**

**Supplemental Figure 1.** Association of HMS donor/recipient status with total estimated volume of shared human milk (S-HM) exchanged among survey respondents (n=167).
